# Effects of Omega-3 Polyunsaturated Fatty Acids, Docosahexaenoic Acid and Eicosapentaenoic Acid, on Post-Surgical Complications in Surgical Trauma Patients: Mechanisms, Nutrition, and Challenges

**DOI:** 10.3390/md22050207

**Published:** 2024-04-30

**Authors:** Asma Ouagueni, Raed M. Al-Zoubi, Ahmad Zarour, Abdulla Al-Ansari, Hiba Bawadi

**Affiliations:** 1 Department of Human Nutrition, College of Health Science, QU-Health, Qatar University, Doha 2713, Qatar; ao1601621@qu.edu.qa; 2 Surgical Research Section, Department of Surgery, Hamad Medical Corporation, Doha 576214, Qatar; ralzoubi@hamad.qa (R.M.A.-Z.); alansari1@hamad.qa (A.A.-A.); 3 Department of Chemistry, Jordan University of Science and Technology, P.O. Box 3030, Irbid 22110, Jordan; 4 Department of Biomedical Sciences, College of Health Science, Qatar University, Doha 2713, Qatar; 5 Acute Care Surgery Division, Department of Surgery, Hamad Medical Corporation, Doha 576214, Qatar; azarour@hamad.qa; 6 Department of Surgery, Division of Urology/Andrology, Hamad Medical Corporation, Doha 576214, Qatar

**Keywords:** omega-3, surgery, post-operative complications, inflammation, trauma

## Abstract

This paper aims to provide an in-depth review of the specific outcomes associated with omega-3 polyunsaturated fatty acids (PUFAs), focusing on their purported effects on post-surgical complications in trauma patients. A comprehensive investigation of omega-3 polyunsaturated fatty acids was conducted until February 2023 using the PubMed database. Surgical trauma is characterized by a disruption in immune response post surgery, known to induce systemic inflammation. Omega-3 PUFAs are believed to offer potential improvements in multiple post-surgical complications because of their anti-inflammatory and antioxidant properties. Inconsistent findings have emerged in the context of cardiac surgeries, with the route of administration playing a mediating role in these outcomes. The effects of omega-3 PUFAs on post-operative atrial fibrillation have exhibited variability across various studies. Omega-3 PUFAs have demonstrated positive effects in liver surgery outcomes and in patients with acute respiratory distress syndrome. Omega-3 is suggested to offer potential benefits, particularly in the perioperative care of patients undergoing traumatic procedures. Incorporating omega-3 in such cases is hypothesized to contribute to a reduction in certain surgical outcomes, such as hospitalization duration and length of stay in the intensive care unit. Therefore, comprehensive assessments of adverse effects can aid in identifying the presence of subtle or inconspicuous side effects associated with omega-3.

## 1. Introduction

Omega-3 polyunsaturated fatty acids (PUFAs), specifically EPA (eicosapentaenoic acid) and docosahexaenoic acid (DHA), are considered essential for human health and exhibit potential therapeutic properties for various illnesses, as demonstrated by numerous scientific investigations. Surgical trauma encompasses a broad disruption in a patient’s post-operative hemodynamic, metabolic, and immunological responses [1]. The term used to describe metabolic instability following trauma is “stress response” [2]. The stress response to surgical trauma bears similarities to the response seen in accidental injuries, because both situations trigger the body’s natural reaction to tissue damage and the initiation of healing processes. Scientists have studied the response to surgery for many years, and based on hemodynamic, metabolic, and hormonal factors, the terminologies “Ebb” and “Flow” were coined by researcher Cuthbertson in 1932 to delineate the phases of traumatic injury [3]. The hypercatabolism and hypermetabolism resulting from the stress response to surgery can lead to undesirable outcomes, including muscle wasting, compromised immune function, an elevated risk of infection, impaired wound healing, organ failure, and even death [4]. Therefore, mitigating the systemic inflammatory, hormonal, and metabolic responses through nutritional supplementation and pharmaceutical interventions is crucial. Nutritional supplementation with anti-inflammatory and antioxidant substances, such as zinc [5], vitamin C [6], omega-3 PUFAs [7], and combinations of these nutrients [8], has indeed shown potential for positive effects on post-surgical trauma and recovery. 

Due to the anti-inflammatory and immunomodulatory properties of omega-3 PUFAs, their use is strongly recommended in cases involving a hyper-inflammatory state, such as surgeries [7,9,10,11,12,13,14,15]. DHA and EPA serve as the foundational components for anti-inflammatory lipid mediators and specific pro-resolving lipid mediators [16]. PUFAs participate in the regulation of the inflammatory response through various reaction pathways. Consequently, increasing omega-3 PUFAs may represent a valuable strategy for guiding the immune response toward the resolution of inflammation [17].

Numerous clinical trials have indeed been conducted to investigate the potential benefits of omega-3 fatty acids, specifically DHA and EPA, as nutraceuticals for improving post-surgical outcomes, particularly in the context of traumatic surgical procedures. These trials have examined various aspects of the effects of omega-3 on surgical patients, including a reduction in inflammation, enhanced immune function, pain management, improved cardiovascular health, positive effects on tissue repair, and a decrease in complications [18,19,20,21,22]. Therefore, this review paper is the first to comprehensively describe the current state of the effect of omega-3 PUFAs on post-surgical complications in trauma patients. Furthermore, it explores the relationship between omega-3 PUFA and post-surgical complications in trauma patients, as well as their effects in the contexts of cardiac surgeries, liver surgeries, and femoral fracture surgeries in hospitalized patients with acute respiratory distress syndrome (ARDS).

## 2. Materials and Methods

The database, PubMed (Medline), was searched for scientific papers published through February 2023. The following key terms were used: (“Fish oil” OR “omega 3” OR “docosahexaenoic acid” OR “eicosapentaenoic acid”) AND (“surgical complications” OR “post-operative complications” OR “surgery induced inflammation” OR “surgical trauma”) AND (“cardiac surgeries” OR “Liver surgeries” OR “Fracture surgeries”).

## 3. Background of Omega-3 Polyunsaturated Fatty Acids

**Figure 1:**
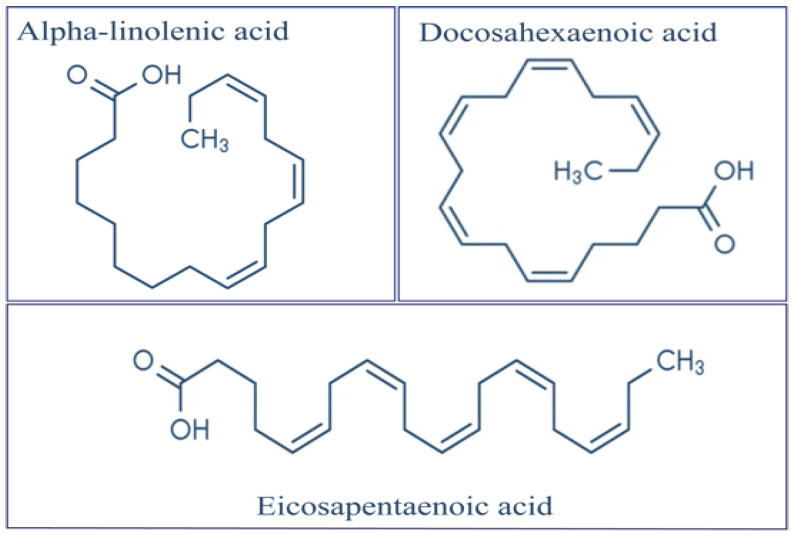
Chemical structures of α-linolenic acid (ALA), docosahexaenoic acid (DHA), and eicosapentaenoic acid (EPA).

Omega-3 PUFAs, often referred to as ω-3, derive their name from their chemical structure, characterized by a commonality: a double bond at the third carbon atom from the methyl (-CH3) end, as depicted in Figure 1. Omega-3 PUFAs encompass a diverse group of fatty acids, with only three molecules of particular importance to the human body: α-linolenic acid (ALA), DHA, and EPA [23]. Considering that ALA cannot be synthesized by the body and is essential for maintaining homeostasis, it must be obtained from dietary sources, such as vegetable oils, nuts, flax seeds, flaxseed oil, green vegetables (primarily in purslane), fruits (predominantly in kiwi), and a limited amount in animal fats (mainly from grass-fed animals) [24]. Consequently, ALA qualifies as an essential omega-3 PUFA [25,26]. The body utilizes the essential omega-3 PUFA (ALA) through a series of chain elongation and desaturation reactions to produce other biologically important metabolites, namely DHA and EPA [27,28]. Despite ALA being the sole essential omega-3 PUFA, it does not convert sufficiently into DHA and EPA, with less than 8% and 4% of the body’s requirements being transformed into EPA and DHA, respectively [29,30]. Therefore, the consumption of dietary-rich sources of DHA and EPA, primarily from seafood sources, can effectively increase their levels [31]. The richest food sources of EPA and DHA omega-3 are found in marine food, particularly in cold-water fatty fish, including anchovies and salmon, which contain EPA and DHA in amounts ranging from 2300 to 2400 mg per 4 oz and 1200 to 2400 mg per 4 oz, respectively [32]. Other seafood options rich in EPA and DHA omega-3 include mackerel, sardines, and trout. These fishes also provide a valuable source of protein and other essential nutrients, making them a healthy addition to any diet.

## 4. Surgical Trauma and Post-Surgical Complications

The systemic response to surgery encompasses a series of interconnected physiological changes that occur in response to a surgical insult [33]. These responses involve a complex interplay of immunological, endocrine, metabolic, and hemodynamic processes. These physiological changes bear similarities to those observed in other types of injuries, such as burns, infections, and traumatic injuries. The response to surgery or other traumas is commonly referred to as the systematic inflammatory response syndrome [34]. The stress response to surgery is typically divided into two phases: the “ebb” and “flow”. The initial “ebb phase” is relatively brief, lasting for approximately 2 to 3 days, and is characterized by reductions in cardiac output, basal metabolic rate, oxygen consumption (VO2), and glucose tolerance. In contrast, the subsequent “flow phase” extends for more than one week, with its duration depending on the severity of the surgery or injury. This phase is characterized by a hypermetabolic and catabolic state, marked by increases in cardiac output, respiratory rate, VO2, hyperglycemia, skeletal muscle catabolism, and a negative nitrogen balance [35]. Notably, the effects of omega-3 can vary based on factors such as the type of surgery, the dosage and duration of substances, and the overall health status of the individual patient. Furthermore, omega-3 is typically considered a part of a comprehensive approach to post-surgical care, which includes other various interventions, such as wound management, pain control, and physical therapy.

### 4.1. Endocrine Response

Afferent stimuli initiate the stress response by transmitting impulses from the site of injury to the hypothalamus, which subsequently activates the hypothalamic–pituitary adrenal axis to restore homeostasis following an injury [35,36,37]. The hypothalamus secretes corticotrophin-releasing hormone (CRH), which stimulates the anterior pituitary gland to release adrenocorticotropic hormone (ACTH) into the bloodstream. In response to ACTH, the adrenal cortex produces glucocorticoids, notably cortisol [33]. During and after surgery, the normal negative feedback mechanism that regulates cortisol secretion by inhibiting CRH and ACTH secretion becomes disrupted, resulting in excessive and continuous cortisol release. Cortisol, being a catabolic hormone, induces hyperglycemia through gluconeogenesis in the liver, negatively affecting surgical outcomes [35]. Moreover, heightened production of growth hormone (GH) by the anterior pituitary gland further increases gluconeogenesis and insulin resistance, while antidiuretic hormone (ADH) from the posterior pituitary gland leads to water reabsorption and reduced renal output. The sympatho-adrenal response results in increased catecholamine release, contributing to hypertension and tachycardia. Surgical trauma also affects other hormones, including insulin, glucagon, and catecholamines, exacerbating the stress response to surgery [35]. Surgical trauma triggers a complex hormonal response that involves the HPA axis, adrenal glands, and various hormones. Disruption of the normal negative feedback loop can lead to prolonged and elevated cortisol levels, thereby influencing metabolism, blood pressure, and other physiological processes. These hormonal changes represent the body’s attempt to respond to the stress of surgery and maintain homeostasis during the healing process. However, excessive or prolonged stress responses can potentially affect surgical outcomes and recovery. Therefore, omega-3 can be valuable for controlling and promoting recovery in such cases.

### 4.2. Immune Response

The magnitude of the immune response is multifaceted, with the severity of trauma, the presence of infection, and the nutritional status of the patient being pivotal factors. This response entails the release of pro-inflammatory and anti-inflammatory cytokines and involves a cell-mediated component [33]. Notably, the production of inflammatory markers, including IL-1; IL-6 tumor necrosis factor-alpha; and acute-phase proteins, such as procalcitonin (PCT) and C-reactive protein (CRP), is massive. An uncontrolled inflammatory response can lead to multiple organ failure or stress-induced organ dysfunction, which is closely associated with post-operative complications, infections, prolonged hospitalization, and increased mortality rates [35]. The immune response to surgical trauma represents a complex interplay of various factors, encompassing the extent of the trauma, the presence of infection, and the nutritional status of the patient. The release of pro-inflammatory and anti-inflammatory cytokines, along with the production of acute-phase proteins, assumes a pivotal role in this intricate process. Effective management of the immune response is imperative to mitigate complications and facilitate a successful recovery following surgery, and omega-3 can play a valuable role in achieving this goal. 

## 5. The Effect of Omega-3 on Traumatic Surgical Outcomes

### 5.1. Coronary Artery Bypass Graft Surgeries

#### 5.1.1. Intravenous Administration

Omega-3 PUFA was administered intravenously in three studies on patients undergoing CABG surgery [38,39,40], as shown in Table 1. The intravenous omega-3 was administered in relatively short durations ranging from 1 day before surgery until discharge. Miliü Veljoviü et al. conducted a study to assess the effects of intravenously administered omega-3 on hematological parameters and platelet activity [38]. They infused a 100 mL lipid emulsion of LCPUFA one day before surgery at four different intervals preceding cardiopulmonary bypass, a surgical procedure known to induce inflammation. The intravenously administered omega-3 in the interventional group did not produce any remarkable effects when compared with the control group receiving 0.9% saline infusion. This lack of significance extended to hematological parameters, transfusion requirements, post-operative blood loss, allogenic red blood cells (RBCs), fresh frozen plasma (FFP), platelet units, post-operative blood loss, and post-operative platelet aggregation ADP test. However, a noteworthy reduction in post-operative platelet aggregation was observed in the n-3 group, as assessed by the COL test. Another study by Mette Berger et al. [39] administered 0.2 g/kg of omega-3 intravenously. They conducted two infusions at 12 and 2 h preoperatively and a final infusion immediately after surgery. In comparison to the saline group, patients receiving n-3 exhibited a substantial increase in the incorporation of EPA and DHA in platelet membranes. In atrial tissue, substantial incorporation of EPA was noted, although no substantial difference was observed in DHA levels. Additionally, a marked improvement in inflammatory markers IL-6 and IL-8, as well as blood glucose levels, was observed. However, no remarkable differences were observed in ICU severity scores, ICU stays, or kidney function. Finally, Heidt et al. [40] discovered the substantial beneficial effect of a 100 mg fish oil infusion/kg for 12 h preoperatively until ICU discharge, focusing on assessing the incidence of post-operative atrial fibrillation lasting more than 15 min. Despite the low dose of the administered omega-3 PUFA, a noteworthy improvement in the occurrence of POAF lasting for more than 15 min was evident. The observed result might be attributed to additional compounds in fish oils known as polar lipids, which, when attached to the polar head groups, are believed to enhance the bioavailability of n-3 PUFA [41,42]. These polar lipids are thought to possess certain cardio-protective properties [42].

#### 5.1.2. Oral Supplementation

Regarding the effects of oral administration of omega-3 on surgical outcomes in patients undergoing coronary artery bypass graft surgery, six studies are summarized in Table 1. Calo et al. [43] found that the oral administration of 2 g of n-3 significantly reduced the incidence of post-operative atrial fibrillation (POAF) lasting more than 5 min (*p* = 0.013) and the length of hospital stay (*p* = 0.017). However, no effect was observed on the episodes of AF (*p* = 0.889). In contrast, a study by Saravanan in 2010 [42], where 2 g/day of orally administered omega-3 was provided in the time interval of 21 to 12 days preoperatively until discharge, found no improvement in the overall incidence of AF (*p* = 0.28), clinical AF (*p* = 0.60), AF burden (*p* = 0.49), hospital stay (*p* = 0.49), and length of stay in ICU/HDH for 1 day (1 to 2 days). Similarly, in Sandesara et al. [44], where patients received 4 g/day of omega-3 preoperatively and 2 g/day post-operatively until the development of atrial fibrillation or until day 14, no improvement was observed in various outcomes, including AF (*p* = 0.67), length of hospital stay (*p* = 0.27), congestive heart failure (*p* = 0.68), myocardial infarction (*p* = 1.00), bleeding requiring reoperation or transfusion (*p* = 0.18), infection (*p* = 0.79), renal failure (*p* = 1.0), respiratory failure (*p* = 1.0), stroke or transient ischemic attack (*p* = 1.0), rehospitalization for AF (*p* = 1.0), readmission to the intensive care unit (*p* = 0.64), and death within 30 days (*p* = 1.0).

Saravanan and colleagues in 2016 [45] examined the effects of omega-3 PUFA on ECG atrial arrhythmic markers and did not reveal any significant effects on ECG P-max duration, POAF (*p* = 0.74), ECG P-wave duration (*p* = 0.25), Cx 40 expression (*p* = 0.40), Cx 43 expression (*p* = 0.44), incidence of AF (*p* = 0.26), or total AF burden (*p* = 0.62). However, omega-3 PUFA exhibited a significant reduction in the duration of atrial fibrillation (*p* = 0.04) and led to shorter ICU stays (*p* = 0.003) and hospital stays (*p* = 0.04) as observed in the study by Vasheghani Farahani et al. [46]. In addition, Sorice et al. [19] reported a noteworthy reduction of 72% in the incidence of AF in the n-3 groups (OR 0.28, *p* = 0.013), with a reduction in AF primarily observed in “on-pump” CABG surgery. However, the length of hospital stay did not exhibit a significant change (*p* = 0.75) in their study. It is noteworthy to say that the dose of the oral omega-3 PUFA ranged from 2–4 g/day to exert an effect while low doses administered intravenously showed an effect in one study which might be due to polar lipids as mentioned earlier.

Elevated concentrations of omega-3 polyunsaturated fatty acids (PUFA) could potentially reduce overall blood pressure [47]. A meta-analysis revealed a J-shaped dose–response curve for omega-3 PUFA, with the most substantial reductions in systolic and diastolic blood pressure observed at moderate doses of docosahexaenoic acid (DHA) and eicosapentaenoic acid (EPA), specifically ranging from 2 g/day to 3 g/day [48]. Moreover, doses above recommended intake of 3 g/day will be associated with further reduction in blood pressure [48]. Additionally, the antithrombotic properties of n-3 fatty acids (FAs) may be linked to an increased risk of bleeding, particularly when high doses of omega-3 PUFA are associated with anticoagulant effects [49,50].

**Table 1:** Summary of RCTs addressing the impact of omega-3 long chain PUFAs on open heart surgeries.

First Author	Sample Size	Duration of Exposure	Control	Time of Measures	Dose	Outcomes	Findings
Intravenous							
Miliü Veljoviü et al., 2013 [38]	40 CABG with the use of CPB	Preoperative 1 day before surgery	0.9% saline infusion	Day before surgery and repeated 4 h before starting CPB (25 mL/h)	100 mL lipid emulsion with a high content n-3 EPA:1.25–2.82 g DHA:1.44–3.09 g	hematological parameters and the activity of platelets	(=) hematological parameters (=) transfusion requirements and post-operative blood loss (=) allogenic red blood cells (RBCs) (*p* = 0.94) (=) fresh frozen plasma (FFP) (*p* = 0.52) (=) platelet units (*p* = 0.73) (=) post-operative blood loss (*p* = 0.356) (=) post-operative platelet aggregation ADP test (*p* = 0.396) (−) post-operative platelet aggregation COL test (*p* = 0.009)
Mette M Berger et al., 2012 [39]	28 undergoing CPB	Perioperative 1 day before	Saline infusion	Blood samples (7 time points) and an atrial biopsy (during surgery), every 1–2 h after surgery	3 infusions of 0.2 g/kg at 12 and 2 h pre-op and immediately after surgery of FO emulsion	Primary outcomes: Incorporation of EPA and DHA into the membrane of circulating platelets and cardiac tissue cells Secondary outcomes: inflammation cardiovascular and organ function severity scores length of mechanical ventilation (ICU) hospital stay safety data	(=) ICU severity score *p* = 0.058 (+) Incorporation of EPA and DHA in platelet membrane (+) EPA in atrial tissue (=) DHA in atrial tissue (−) IL-6 (*p* = 0.018) (−) IL-8 (*p* = 0.005) (=) IL-10 (*p* = 0.10) (=) ICU stay (*p* = 0.118) (=) kidney function (plasma carnitine and plasma urea) (−) average glycemia (*p* < 0.0001)
Heidt et al., 2008 [40]	CABG	Perioperative 12 h before surgery until discharge from ICU	100 mg soya oil/kg/day	Standard 12-lead ECG was performed daily from admission to hospital until transfer	100 mg fish oil/kg body weight/day	POAF > 15 min	(−) POAF
Oral							
Calo et al., 2005 [43]	160	Perioperative 5 days before surgery until discharge	Standard care	first four to five post-operative days. 4 weeks follow up after discharge	2 gelatin capsules/day 2 g/day 850–882 mg EPA and DHA	POAF >= 5 min Hospital length of stay	(−) POAF (*p* = 0.013)) (=) episodes of AF (*p* = −0.889) (−) length of hospital stay (*p* = 0.017)
Saravanan et al., 2010 [51]	108	Perioperative 12–21 days median 16 days	2 g/day Olive oil	12–21 days presurgical until Discharge	2 g/day 85% to 88% EPA_DHA as ethyl esters	POAF >= 30 s Clinically recognized AF AF burden Length of hospital stay ICU stay	(=) Overall incidence of AF (*p* = 0.28) (=) Clinical AF (*p* = 0.60) (=) AF burden (*p* = 0.49) (=) hospital stay (*p* = 0.49) (=) Length of stay in ICU/HDH 1 day (1 to 2 days)
Sandesara CM et al., 2012 [44]	260	Perioperative 2 days before	Matched placebo (corn oil)	Day of screening, day of surgery and post operative day 4	Capsules Each 1 g capsule of n3-PUFA contained ≥ 900 mg of n-3 ethyl esters (465 mg EPA and 375 mg DHA) Pre op. 4 g/day Post op. 2 g/day until AF or unitl 14 days	Any episode of AF	(=) AF (*p* = 0.67) (=) Legth of hospital stay (*p* = 0.27) (=) Congestive heart failure (*p* = 0.68) (=) Myocardial infarction (*p* = 1.00) (=) Bleeding requiring reoperation or transfusion (*p* = 0.18) (=) Infection (*p* = 0.79) (=) Renal failure (*p* = 1.0) (=) Respiratory failure (*p* = 1.0) (=) Stroke or transient ischemic attack (*p* = 1.0) (=) Rehospitalization for AF 1 (*p* = 1.0) (=) Readmission to intensive care Unit (*p* = 0.64) (=) Death within 30 d (*p* = 1.0)
Vasheghani Farahani et al., 2017 [46]	478	Perioperative 5 days preoperative until discharge	Olive oil soft gelatin capsules	Not specified	2 g/day Soft gelatin capsules 300 mg EPA 200 mg DHA	POAF until 96 h during ICU and post ICU Length of hospital stay	(=) Incidence of POAF (*p* = 0.07) (−) Duration of AF (*p* = 0.04) (=) Episodes of AF (*p* = 0.06) (−) ICU stay (*p* = 0.003) (−) Hospital stay (*p* = 0.04)
Sorice, M. et al., 2011 [19]	201	5 days preoperatively –discharge	control	Not specified	gelatinous capsules EEPA and DHA ethyl esters 850–882 EPA	POAF incidence Impact of surgical technique on incidence of POAF Length of post-operative hospital stay	(−) AF incidence in n-3 groups (OR 0.28 (*p* = 0.013) (=) Length of hospital stay (days) (*p* = 0.75) (−) AF in “on-pump” CABG, no benefit to off pump groups
Saravanan et al., 2016 [45]	61	14 days preoperatively	Olive oil	Baseline and during surgery	2 g/day (Omacor capsules) 760 mg DHA 920 mg EPA	ECG atrial arrhythmic markers	(=) ECG P-max duration POAF (*p* = 0.74) (=) ECG P-wave duration (*p* = 0.25) (=) Cx 40 expression (*p* = 0.40) (=) Cx 43 expression (*p* = 0.44) (=) Incidence of AF (*p* = 0.26) (=) Total AF barden (*p* = 0.62)

ECG: electrocardiogram, PCR: polymerase chain reaction, Cx40: Connexin 40, Cx43: Connexin 43, AF: atrial fibrillation, FO: fish oil, CPB: cardiopulmonary bypass, ICU/HCU: intensive care/high dependency, (+): Significant increase, (−): Significant decrease, (=): No Significant effect.

### 5.2. Gastrointestinal and Liver Surgeries

A meta-analysis conducted in May 2020 on patients who underwent liver tumor surgery revealed that omega-3 was considerably effective in reducing the incidence of post-operative infections [13]. However, a reduction in the risk of complications was observed only when omega-3 was continuously provided in the perioperative period, rather than in the pre- or post-operative period alone. Additionally, no effects were observed in the cases of mortality, liver failure, biliary leakage, bleeding, or ileus [13]. The methodologically strongest and most recent RCT (*n* = 261 patients) included in the meta-analysis, conducted by Linecker et al. [16], revealed that the intravenous administration of omega-3 perioperatively (100 mL Omegaven) failed to confer protection against post-operative complications after liver surgery. This finding suggests that alleviating inflammation alone may not be sufficient to achieve positive results in reducing the incidence of post-surgical complications following liver surgery [15]. Another RCT study, conducted by Gong et al., assessed the effectiveness of Omega-3 in treating patients after hepatectomy. The findings demonstrated that omega-3 PUFAacid-based lipid emulsions for treating patients after hepatectomy are safe and effective in controlling inflammation, protecting liver function, and reducing the rate of total complications and the length of hospital stay [52]. Moreover, experimental studies have shown that omega-3 PUFAs effectively reduce severe hepatic steatosis and protect the liver from ischemia-reperfusion injury, which is associated with improved liver regeneration and functional recovery following hepatectomy [53].

When examining the effect of omega-3 on liver status in patients undergoing bariatric surgery, omega-3 can be beneficial, similar to a low-calorie diet (800 kcal), in reducing liver steatosis before bariatric surgery, particularly in morbidly obese women [54]. Both groups showed reductions in overall liver volume (total liver volume, volume of the left liver lobe, and visceral fat area, but the low-calorie diet group exhibited more considerable reductions, albeit with a higher incidence of complications) [54]. These remarkable reductions in the aforementioned outcomes suggest that presurgical Omega-3 may offer a promising alternative to a low-calorie diet. This approach is considered more patient-friendly, because it is less restrictive and allows for more liberal food consumption (2000 kcal + 2 g omega-3/day). It can serve as a safe substitute for a low-calorie diet, which, due to its restrictive nature, may lead to lower patient compliance and a state of malnutrition before surgery, potentially resulting in more side effects. The observed effect is likely attributed to the anti-inflammatory properties of omega-3, which help alleviate the low-grade inflammation associated with obesity. 

In a recent systematic review investigating the impact of preoperative omega-3 fatty acid supplementation on inflammatory responses and clinical outcomes in major gastrointestinal surgery, the findings suggest a lack of substantial evidence to recommend the routine use of such supplementation, even when continued post-operatively [55]. The administered treatments, involving combinations of eicosapentaenoic acid (EPA) and docosahexaenoic acid (DHA), or EPA alone, did not reveal significant differences in mortality rates. Hospitalization durations exhibited variability, and the effects on inflammatory markers were inconclusive [55].

A parallel meta-analysis corroborated these results, indicating that omega-3 polyunsaturated fatty acids (PUFAs) demonstrated no significant impact on the post-operative inflammatory response in patients undergoing abdominal surgeries, despite the observed reduction in inflammatory markers [56]. These collective findings emphasize caution in endorsing preoperative omega-3 fatty acid supplementation for major gastrointestinal surgery.

### 5.3. Omega-3 and Femoral Fracture Surgeries

The available data regarding the effectiveness of omega-3 in improving post-surgical outcomes following femoral fracture surgeries are limited. One of the most common complications associated with femoral fracture surgeries is deep vein thrombosis (DVT). Only one RCT (*n =* 452 elderly participants (intervention—226 and control—226)) has investigated the relationship between post-surgical risk of DVT and pulmonary embolism following proximal femoral fractures. The authors of this study concluded that providing oral omega-3 on a regular basis can effectively reduce the risk of post-surgical pulmonary embolism, venographic events, and symptomatic DVT [10]. The potential mechanism of action for this effect could be attributed to the antithrombotic properties of EPA. In a rat tail thrombosis model, oral EPA intake was evaluated for its effect on venous thromboembolism, and the results indicated that omega-3 remarkably reduced the lesion area in the rat tail, demonstrating strong antithrombotic effects following EPA dosing [57]. Furthermore, recent longitudinal studies have revealed a negative correlation between higher intake of omega-3 from sea sources and high serum omega-3 levels with mortality and venous thromboembolism rates in the elderly population [58,59]. Therefore, while omega-3 may hold potential benefits for other surgical procedures or medical conditions, its specific role in femoral fracture surgery outcomes, particularly in preventing DVT, may require further research and clinical trials to establish its effectiveness and safety. 

### 5.4. Omega-3 PUFA Supplementation in Hospitalized Patients with ARDS

Patients suffering from ARDS have reported improvements after receiving omega-3 PUFAs. ARDS is a severe respiratory condition characterized by widespread inflammation in the lungs, which can lead to life-threatening respiratory failure. A recent meta-analysis encompassing 12 randomized controlled trials (*n =* 1280 patients) conducted in patients with ARDS revealed that omega-3 PUFA supplementation was associated with enhancements in the PaO2/FiO2 ratio. Additionally, statistical trends indicate shorter ICU stays and reduced durations of mechanical ventilation. However, the incidence of infectious complications remained unchanged. In a study solely focusing on enteral omega-3 PUFA supplementation, a statistically significant reduction in ARDS mortality was observed [9]. A Cochrane meta-analysis involving 10 studies further substantiated the positive effects of omega-3 PUFAs, demonstrating a reduction in ARDS mortality compared with a lipid-rich enteral formula. Nonetheless, due to remarkable variations between trials, it remains uncertain whether omega-3 PUFA supplementation can consistently influence mortality rates, oxygenation levels, durations of mechanical ventilation, and lengths of ICU stays [60].

Gupta et al. [61] conducted a RCT involving 61 ventilated patients with ARDS to precisely investigate the benefits of intravenous omega-3 PUFA emulsion. The study revealed that while the primary outcome, which assessed changes in respiratory parameters, did not show significant alterations in patients with ARDS, the decline in the PaO2/FiO2 ratio from baseline to day 14 was significantly greater in the control group compared with the patients treated with intravenous omega-3 PUFA emulsion. Furthermore, the subsequent trial indicated a trend toward improved survival in the omega-3 PUFA emulsion group (77%) in contrast to the control group (56%). Importantly, the study also found that lipid emulsions enriched in omega-3 PUFAs are safe for use in individuals with ARDS and do not have deleterious effects when administered intravenously [17]. This remarkable finding underscores the potential benefits of omega-3 PUFA supplementation for individuals with ARDS.

## 6. Mechanisms of Action

### 6.1. Omega-3 as an Anti-Inflammatory Molecule

The omega-3-related immunity and inflammatory response reactions are modulated by DHA, mainly with the participation of EPA by using different proposed mechanisms. The first mechanism is by being a precursor for some cell signaling lipid mediators that function as pro-resolving mediators or specialized pro-resolving mediators (SPMs). These SPMs include resolvins, protectins, and docosatrienes [62]. Their role is characterized by the initiation of inflammation termination or resolution for the restoration of homeostasis [63]. Two classes of resolvins are grouped according to their precursors, either from EPA, known as E-series (RvE), or DHA, known as D-series (RvD) resolvins [64]. The formation of RvE from EPA can be enhanced by the acetylated cyclooxygenase-2 (COX-2) and 5-lipoxygenase (5-LOX) and Cytochrome P450 [64,65]. The enzymatic conversion of omega-3 polyunsaturated fatty acids into SPMs actively disrupts inflammatory circuits and skews the immune response toward repair and homeostasis [66]. Additionally, certain SPMs suppress viral replication and ameliorate the severity of viral pneumonia in experimental models [67,68]. However, DHA has much wider roles in terminating inflammation through the formation of much wider ranges of pro-resolving molecules, including D-series resolvins, protectins, and maresins, where the latter is a macrophage mediator in resolving inflammation (MaR1) [69,70,71]. The macrophage mediators resolve inflammation by promoting the formation of macrophages through monocyte differentiation that leads to phagocytosis and down-regulating inflammation. These macrophages are activated by the lipid mediators produced from DHA and EPA that has been mentioned previously.

**Figure 2:**
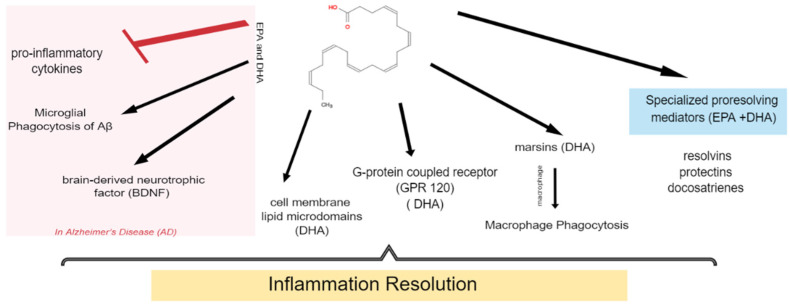
Summary of inflammatory resolution roles of EPA and DHA through different mechanisms.

Moreover, researchers have discovered that DHA can reduce inflammation by stimulating the G-protein-coupled receptor (GPR 120), which is a cell membrane receptor specific to DHA [72]. Additionally, promoting inflammation resolution may involve the alteration of cell membrane lipid microdomains, which play a crucial role in inflammation-related cell signaling pathways [73]. DHA can reduce the activity of Toll-like receptor 4 (TLR4), a pro-inflammatory molecule, thus contributing to the resolution of inflammation [74]. Furthermore, omega-3 fatty acids have down-regulatory effects on pro-inflammatory cytokines and stimulate the production of neuroprotective brain-derived neurotrophic factor (BDNF), offering a promising avenue for AD treatment [75]. Figure 2 summarizes the proposed mechanisms of action of omega-3 molecules (DHA and EPA) in resolving inflammation.

**Figure 3:**
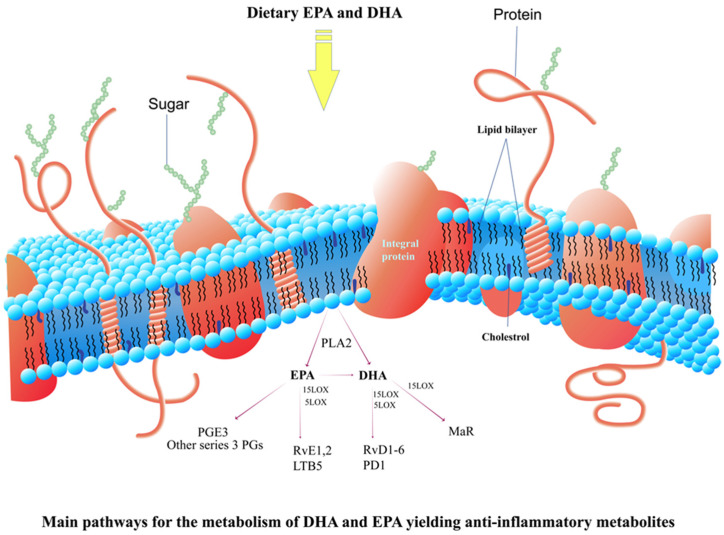
The principal metabolic pathways for docosahexaenoic acid and eicosapentaenoic acid that generate anti-inflammatory metabolites.

The two primary polyunsaturated fatty acids (PUFAs), namely docosahexaenoic acid DHA and EPA, can undergo two separate procedures in order to achieve their release from cellular membranes (Figure 3). One mechanism involves the action of phospholipase A2 (PLA2), which allows the liberation of DHA and EPA from the cell membrane. As an alternative, dietary intake of DHA and EPA can provide a source for enzymatic conversion through the activity of lipoxygenase (LOX) and cyclooxygenase (COX) enzymes. These enzymatic reactions result in the production of bioactive compounds that have anti-inflammatory effects. The metabolites of these substances connect to the receptors that are specific to them and activate anti-inflammatory properties in tissues, primarily via restructuring the transcriptome. In combination, these multiple impacts result in reductions in IL-6, IL-1, or TNFα, which are crucial cytokines that incite cytokine storm.

### 6.2. Omega-3 in Trauma

In cases of inflammation related to trauma, such as stroke, traumatic brain injury (TBI), or spinal cord injury (SCI), the balance of free fatty acids becomes disrupted, leading to their accumulation in the cerebrospinal fluid (CSF). In the context of TBI and SCI, the pathophysiology of these conditions begins with the disruption of the blood–brain barrier (BBB) due to physical damage to the skull. This disruption further compromises essential functions, resulting in a state of reduced oxygenation and ultimately leading to apoptosis or cell death [76,77,78]. The dead cells trigger excitotoxicity in the surrounding undamaged cells, caused by the influx and excessive release of glutamate, a major excitatory neurotransmitter, into the healthy cells [79,80]. This, in turn, activates calcium-dependent lipases, including cytosolic phospholipase A2 (cPLA2), due to the increased influx of Ca^2+^ through glutamate receptors and voltage-gated Ca^2+^ channels [81,82]. Consequently, the deacylation–reacylation reactions are disrupted due to the overactivation of phospholipase A2 (PLA2) and low levels of ATP [82,83]. This disruption leads to a significant release of free fatty acids, including arachidonic acid (AA) and DHA, from the cell membranes.

The overactivation of cPLA2α is also known to mediate inflammatory reactions through certain cell receptors, leading to the activation of mitogen-activated protein kinase (MAPK) and protein kinase C (PKC), ultimately resulting in the release of AA. AA serves as a substrate for enzymes such as cyclooxygenases (COX1 and COX2) and lipoxygenase (LOX), and it also acts as a precursor for various inflammation mediators, including eicosanoids. These processes culminate in the promotion of inflammation within the cells and tissues [82,84].

**Figure 4:**
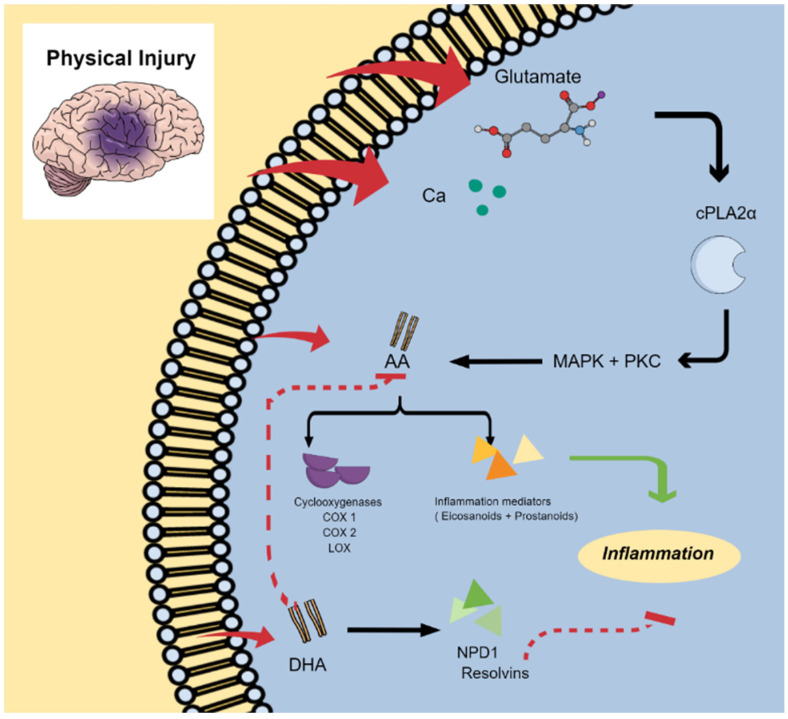
Summary of induced inflammation in the CNS and the role of omega-3 PUFA (DHA) in the inflammation resolution process.

The resolution of inflammation is primarily promoted by the key molecule DHA. DHA is renowned for its anti-inflammatory and neuroprotective actions, because it serves as a precursor for neuroprotectin D1 (NPD1) and resolvins, which are mediators that contribute to the down-regulation of inflammation [85,86,87,88]. Furthermore, the release of DHA is associated with calcium-independent iPLA2β, which some studies suggest counters the inflammatory processes promoted by calcium-dependent cytosolic PLA2 (cPLA2α) [85,86]. Finally, because the enzymatic reactions of omega-3 and omega-6 fatty acids share the same enzymatic pathways, increasing the concentration of omega-3 fatty acids can be a proposed method for resolving inflammation by competing with omega-6 fatty acids for enzymes, thus reducing the conversion of AA into pro-inflammatory eicosanoids and subsequently dampening inflammatory responses (Figure 4) [89].

### 6.3. Omega-3 in Wound Healing

Ekci et al. [90] conducted experiments involving colon anastomoses in rats and demonstrated that the post-operative anastomotic bursting strength was considerably higher in animals receiving preoperative dietary supplementation with a combination of ascorbic acid and fish oil. Bursting strength also improved in animals supplemented with either ascorbic acid or omega-3 fatty acids alone. In contrast, Albina et al. [91] fed animals a diet containing fish oil for 21 d before surgical incision was made and for 10 or 30 d after wounding. They showed that collagen content was similar at 10 and 30 days, but 30-day wounds were evidently weaker in animals treated with fish oil compared with animals receiving corn oil.

McDaniel et al. [92,93] conducted a study to investigate the effects of fish oil administration compared to mineral oil administration in healthy adults. The participants, aged 18 to 45, were randomly assigned to receive either EPA (EPA) and DHA per day or mineral oil per day, along with low-dose aspirin. Eight-millimeter blisters were induced on the participants’ nondominant arm. As expected, the analysis of plasma fatty acid profiles showed a significant shift toward omega-3 fatty acids, along with a notable decrease in the omega-6/omega-3 fatty acid ratio. On the fifth day, the group receiving the omega-3 supplement exhibited greater blister epithelialization compared with the control group. Additionally, a shift in plasma eicosanoids toward the EPA profile was observed, indicating improved wound healing.

## 7. Limitations, Summary, and Conclusions

In conclusion, multiple studies suggest that the use of omega-3 PUFAs is highly recommended in cases of hyper-inflammatory conditions, such as surgeries, given their anti-inflammatory and immunomodulatory properties. ASPEN recommends enriching parenteral nutrition with omega-3 because it reduces infection rates, ICU and hospital stays, and inflammatory markers. The available evidence indicates that omega-3 PUFAs can have beneficial effects on various surgical outcomes, including coronary artery bypass graft surgeries [39,40,43], post-surgical deep vein thrombosis following femoral fracture surgeries [10], and liver surgeries [15,52,53]. Notably, the effectiveness of omega-3 depends on the type of surgery, dosage, timing, and route of administration. Moreover, long-term administration of small doses of omega-3 through enteral or oral intake appears to be more effective in reducing post-operative atrial fibrillation (POAF) in coronary artery bypass graft surgeries compared with short-term administration through parenteral nutrition. These might be due to the polar lipids which increase the bioavailability of omega-3 PUFA and have cardioprotective roles [41,42]. In the case of liver surgeries, perioperative omega-3 administration has been associated with a reduction in infection incidence in liver tumor surgeries, as well as improvements in inflammation, complication rates, hospital stays, and liver functionality in patients undergoing hepatectomy. Additionally, omega-3 has shown promise in reducing liver steatosis and volume prior to bariatric surgery and improving the high inflammatory status in ARDS.

The effects of omega-3 PUFAs can vary from one RCT to another due to differences in study designs, study populations, duration of supplementation, and outcome variables. Additionally, variations in the dosage and composition of omega-3 PUFAs used in each study can lead to different observed outcomes, making it important to consider these factors when interpreting study results. 

Although ample literature discusses the effects of omega-3 on various surgical outcomes, the exact mechanisms by which omega-3 PUFAs improve surgical outcomes remain unknown. Researchers continue to investigate this area to gain a deeper understanding. Furthermore, the majority of RCTs have focused on long-chain omega-3 PUFAs, specifically DHA and EPA, in their studies. However, medium-chain omega-3 alpha-linolenic acid (ALA), despite its known anti-inflammatory and antioxidant properties, has received relatively little attention in the field. Therefore, it is suggested that further clinical interventions be conducted to assess the role of the ALA omega-3 PUFA in preventing post-surgical complications. Moreover, there is an ongoing study over the last 5 years on bioactive polar lipids containing EPA, DHA, and ALA within their structures and evidence regarding PL supplementation, although promising, is limited and further research is required.
